# Substrate-anchored and degradation-sensitive anti-inflammatory coatings for implant materials

**DOI:** 10.1038/srep11105

**Published:** 2015-06-16

**Authors:** Duo Wu, Xingyu Chen, Tianchan Chen, Chunmei Ding, Wei Wu, Jianshu Li

**Affiliations:** 1 College of Polymer Science and Engineering, Sichuan University, Chengdu 610065, China; 2 State Key Laboratory of Polymer Materials Engineering, Sichuan University, Chengdu 610065, China

## Abstract

Implant materials need to be highly biocompatible to avoid inflammation in clinical practice. Although biodegradable polymeric implants can eliminate the need for a second surgical intervention to remove the implant materials, they may produce acidic degradation products *in vivo* and cause non-bacterial inflammation. Here we show the strategy of “substrate-anchored and degradation-sensitive coatings” for biodegradable implants. Using poly(lactic acid)/hydroxyapatite as an implant material model, we constructed a layer-by-layer coating using pH-sensitive star polymers and dendrimers loaded with an anti-inflammatory drug, which was immobilised through a hydroxyapatite-anchored layer. The multifunctional coating can effectively suppress the local inflammation caused by the degradation of implant materials for at least 8 weeks *in vivo*. Moreover, the substrate-anchored coating is able to modulate the degradation of the substrate in a more homogeneous manner. The “substrate-anchored and degradation-sensitive coating” strategy therefore exhibits potential for the design of various self-anti-inflammatory biodegradable implant materials.

Implant materials have been widely applied in clinical practice with biocompatibility requirements such as anti-inflammation^1^,^2^,^3^,^4^,^5^,^6^,^7^. Both second- and third-generation biomedical materials are designed such that they possess biodegradability, thereby avoiding a second surgical intervention to remove the materials^8^. However, the most commonly used biodegradable polymeric materials, such as poly(lactic acid) (PLA), poly(glycolic acid) (PGA), poly(ɛ-caprolactone) (PCL), and their copolymers, can degrade into acidic products *in vivo*, which may arouse non-bacterial inflammation^9^,^10^. Organic/inorganic hybrid materials composed of biodegradable polymers and bioactive particles have been intensively studied in surgical reconstruction and bone tissue engineering^11^,^12^,^13^,^14^ to mimic the composition of natural bone (mineral/collagen fibrils)^15^. Poly(lactic acid)/hydroxyapatite (PLA/HA) composite material is an elegant example of bone-related medical application, whereby the biodegradable PLA is resorbable and can easily be processed while the bioactive HA is osteoconductive and has bone bonding capability^16^,^17^,^18^,^19^,^20^,^21^,^22^,^23^. The incorporation of alkaline HA can partially neutralise the acid produced by PLA, but it cannot completely avoid the decrease in pH of peripheral tissues^17^,^21^. Also, it is well-known that local inflammation can result in acidosis *in vivo*^24^,^25^,^26^. Thus, variations in pH caused by the degradation of implanted polymers and inflammatory tissue can be applied as a stimulus to design smart anti-inflammatory surfaces for implant materials.

In the past decades, layer-by-layer (LbL) self-assemblies have been widely investigated as surface coatings of biomedical materials^2^,^7^,^13^,^27^,^28^,^29^. A variety of components such as drugs can be incorporated into LbL films in a precise manner. Particularly, pH-sensitive LbL coatings can release drugs based on the pH-induced decomposition or changes in the permeability of the LbL building blocks^30^. Moreover, it has been found that hyperbranched polymers, such as star polymers and dendrimers, could be applied as LbL building blocks^31^,^32^,^33^,^34^,^35^,^36^. The LbL systems constructed by hyperbranched polymers have stimuli-responsive behaviours that are superior to those of their linear counterparts because of their unique conformations^33^,^35^. In our previous study, we fabricated a series of pH-sensitive LbL multilayer films based on positively charged star-poly[2-(dimethylamino) ethyl methacrylate] (star-PDMAEMA)^37^,^38^,^39^,^40^,^41^ and synthesised a type of HA-anchored alendronate-conjugated poly(amido amine) dendrimer (ALN-PAMAM-COOH)^42^,^43^. Dendrimers can also significantly enhance the solubility of hydrophobic drugs by covalently conjugating with drugs or forming a non-covalent drug inclusion complex^44^,^45^,^46^,^47^, and thus it is reasonable to load anti-inflammatory drugs into dendrimers and then apply them as LbL building blocks. Herein, we demonstrated the design of a substrate-anchored and degradation-sensitive anti-inflammatory LbL coating for implant materials, which can significantly reduce inflammation for 8 weeks after *in vivo* implantation.

In the present study, PLA/HA composite was prepared as a typical implant material. It was then coated with multifunctional LbL films with HA-anchored and pH-sensitive properties to effectively suppress the local inflammation of peripheral tissues caused by the acidic degradation products of PLA (Fig. 1). Indomethacin (IND) was chosen as a model of anti-inflammatory drug and was loaded into the PAMAM-COOH dendrimer. ALN-PAMAM-COOH was applied as the first layer on the PLA/HA surface since the ALN moiety could tightly immobilise the LbL multilayers on the substrate. Then, six bilayers of IND-loaded PAMAM-COOH/star-PDMAEMA were fabricated on top of the first bilayer (ALN-PAMAM-COOH/star-PDMAEMA). When the implanted PLA/HA substrate inevitably degrades in human body, it produces acidic degradation products and causes a decrease in pH in the microenvironment. In this case, the LbL films coated on the surface exhibit a substrate degradation-sensitive release profile of IND to suppress local inflammation. The facile strategy of preparing multifunctional coatings with substrate-anchored and degradation-sensitive anti-inflammatory properties justifies its broad biomedical applications.

## Results and discussion

We prepared IND-loaded PAMAM-COOH as one of the building blocks of the LbL coatings. The inclusion of IND in PAMAM-COOH was confirmed by ^1^H NMR (Supplementary Figure S1). Compared with PAMAM-COOH alone, peaks corresponding to IND were clearly observed in the spectrum of IND-loaded PAMAM-COOH. H-a shows a single peak in DMSO-d_6_ but appears as two separated peaks in D_2_O. In addition, the proportion of the integral area of protons assigned to different parts of IND has been changed slightly, e.g., the ratio of H-a to H-e changed from 4:3 to 4:2.82, which suggests that the indole ring near the carboxyl group was to some extent shielded by PAMAM-COOH. Additionally, the new peaks and the shifts in the characteristic peaks of IND-loaded PAMAM-COOH in Fourier transform infrared spectroscopy (FTIR, Supplementary Figure S2) measurements and the change in endothermal transition peaks in differential scanning calorimetry (DSC, Supplementary Figure S3) provide further evidences of the successful formation of IND-loaded PAMAM-COOH. The amount of IND loaded into PAMAM-COOH was concentration-dependent (Supplementary Figure S4). The solubility of IND increased linearly at the beginning, when the concentration of PAMAM-COOH was lower than 1.63 mg mL^−1^ (stage I in Supplementary Figure S4). At this stage, IND was mainly encapsulated in the cavity of each isolated dendrimer. When the concentration of the dendrimer increased, the aggregation of the dendrimers in water allowed more IND to be encapsulated not only in the interior cavity of the dendrimers but also in the aggregated dendrimer network (stage II). With further increase in the concentration of the dendrimer, the IND loading profile may be further changed because of the increased electronic interactions between the dendrimers (stage III). However, in order to establish the experimental design in simpler conditions, we chose a concentration of 1 mg mL^−1^ (within the first linear stage) for further LbL fabrication and characterisations since IND was considered to be mainly included in the interior of the dendrimer at this condition.

**Figure 2:**
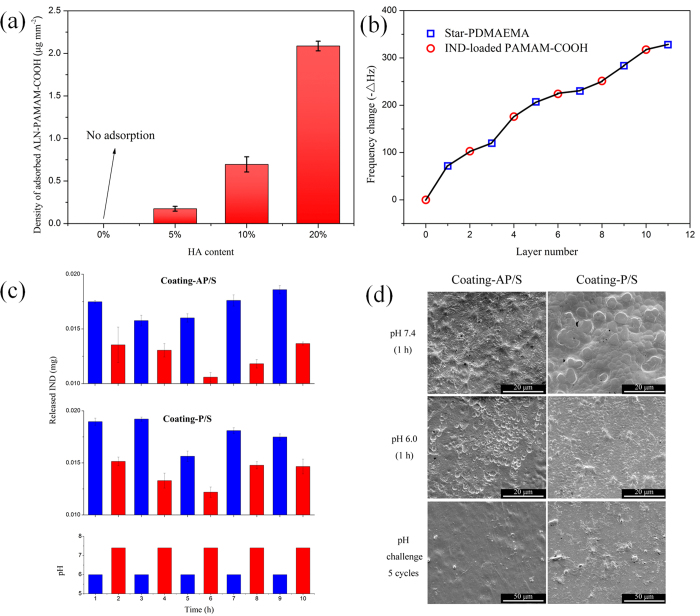
Fabrication of the LbL coatings and their pH-sensitive properties. (**a**) The density of ALN-PAMAM-COOH adsorbed onto the PLA/HA substrate with different HA contents (n = 3). (**b**) QCM frequency changes as a function of layer number for star-PDMAEMA/IND-loaded PAMAM-COOH bilayers. Each solution was prepared as 1 mg mL^−1^ at pH 6. (**c**) Repeated up-down release of IND from Coating-AP/S and Coating-P/S in response to stepwise pH challenge between 6.0 and 7.4 (n = 3). (**d**) SEM images of the surface morphologies of Coating-AP/S and Coating-P/S after being immersed in PBS at pH 7.4 for 1 h, in PBS at pH 6.0 for 1 h, and stepwise pH challenge between pH 6.0 and 7.4 for 5 cycles (images taken after pH 7.4).

To quantify the amount of ALN-PAMAM-COOH adsorbed on PLA/HA substrates with different HA contents, we calculated the adsorption density by measuring the amount of ALN-PAMAM-COOH retained after thoroughly washing the substrates (Fig. 2a). It is obvious that the adsorption density of ALN-PAMAM-COOH on PLA/HA increases with the increase in HA content. As can be seen, almost no ALN-PAMAM-COOH was retained on the surface of the pure PLA substrate (HA content: 0%) after washing. PLA is hydrophobic, and hence it disrupts the adsorption of the hydrophilic ALN-PAMAM-COOH on its surface. On the other hand, since the ALN moiety has HA-binding specificity, ALN-PAMAM-COOH in aqueous solution would adsorb onto HA. The binding strength is so strong that ALN-PAMAM-COOH would still be retained after washing. The visible adsorption of FITC-labelled ALN-PAMAM-COOH on PLA/HA also confirms the increasing adsorption density with the increase in HA content (Supplementary Figure S5). The above results are consistent with those of our previous study, whereby ALN-PAMAM-COOH strongly bound to human tooth enamel (mainly HA)^42^. Thus, it is reasonable to apply ALN-PAMAM-COOH as the first layer to construct a substrate-anchored LbL coating for implant materials made of PLA/HA composites.

PLA/HA substrate with 10% HA was chosen as a typical substrate for further LbL fabrication. The sequential depositions of the LbL coatings were verified both by UV/vis adsorption spectroscopy and quartz crystal microbalance (QCM). There was an obvious alternative adsorption and desorption behaviour of PAMAM-COOH in alternating deposited LbL coatings (Supplementary Figure S6). The absorbance decreased after each star-PDMAEMA layer was deposited. The aqueous star-PDMAEMA solution also became whitish after manufacturing several layers. The reason is that the PAMAM-COOH extracted from the LbL coatings could form electrostatic complexes with the star-PDMAEMA in solution^37^. The LbL assembly processes between the oppositely charged star-PDMAEMA and PAMAM-COOH (Supplementary Figure S7) or IND-loaded PAMAM-COOH (Fig. 2b) on the PLA/HA substrate were also monitored by QCM. Electrostatic attraction was the dominant driving force of the construction process. The resonance frequency (F) was decreased when the resonator was exposed to each solution. Since –ΔF is related to the adsorbed mass, the trend suggests that these building blocks have been successfully deposited layer by layer on the substrate. The UV/vis adsorption spectroscopy and QCM measurements together confirm the successful fabrication of IND-loaded PAMAM-COOH/star-PDMAEMA LbL coatings.

For comparison, two types of LbL coatings were fabricated on the surface of the PLA/HA substrate (HA: 10%) in the form of {PAMAM-COOH/star-PDMAEMA + (IND-loaded PAMAM-COOH/star-PDMAEMA)_6_} and {ALN-PAMAM-COOH/star-PDMAEMA + (IND-loaded PAMAM-COOH/star-PDMAEMA)_6_}, which were denoted as Coating-P/S and Coating-AP/S, respectively. The theoretical amount of IND loaded in these coatings was 0.288 mg according to the complexation ratio of IND in PAMAM-COOH at 1 mg mL^−1^ (Supplementary Figure S4).

Since PLA degradation and tissue inflammation can cause a decrease in the pH of the tissues adjacent to the implant materials, the pH-sensitive release profiles of IND were evaluated *in vitro* to study the self-regulated IND release mechanism from the LbL coatings. Both Coating-P/S and Coating-AP/S released more IND at pH 6.0 than at pH 7.4 during the whole process (Supplementary Figure S8), reflecting the pH sensitivity of these LbL coatings. In addiion, both coatings showed faster release profiles at the beginning and slowed down later. These LbL coatings still possessed the ability to release IND even at normal physiological conditions (pH 7.4), which could be ascribed to the swelling of the coatings in solution. These phenomena are beneficial for implant materials because they need to be able to suppress acute inflammation at the injury sites right after implantation surgery. It should be noted that the *in vitro* release test was performed in an accelerated condition with a large volume of solution, and thus the release time *in vitro* is only related to but not exactly the same that *in vivo*.

To further evaluate pH sensitivity, stepwise pH challenges were exerted on the LbL coatings by immersing them alternately into PBS solutions at pH 6.0 and 7.4. Both Coating-P/S and Coating-AP/S exhibited obvious “up” and “down” IND release profiles in response to changes in pH (Fig. 2c). Their sensitivities were caused by the permeation of H^+^ into the LbL coatings constructed by star polymers and dendrimers. Star-PDMAEMA is a weak polyelectrolyte that can display pH-responsive behaviours in solution, which are reflected by reversible changes in size^35^,^37^. The arms of star-PDMAEMA can be highly protonised and more stretched with decreasing pH, which can in turn change the density and permeability of the LbL coatings^48^. Consequently, the release of IND from the LbL coatings could be faster at a lower pH. It is interesting to see that the “up-down” release profile of Coating-AP/S was more stable than that of Coating-P/S during the last three pH-challenge cycles, which was probably due to the better substrate-anchoring property of ALN-PAMAM-COOH in the first layer of Coating-AP/S than that of PAMAM-COOH in the first layer of Coating-P/S.

To elucidate the difference in pH sensitivity, the surface morphology transitions of the LbL coatings were observed by SEM (Fig. 2d). Without any treatment, the fabricated LbL coating was rather smooth and continuous (Supplementary Figure S9). The coatings became slightly rough and discontinuous after being immersed in PBS at pH 7.4, which may have been due to their swelling in solution (Fig. 2d). Moreover, they presented rougher surface morphologies after immersion in PBS at pH 6.0, which may have been due to the structural rearrangement of the star-PDMAEMA layer^35^,^37^,^38^. The microporous structure shown on the LbL coatings indicates that the decrease in pH led to certain changes in their interior structure. The large microsized pores, especially in Coating-AP/S, were caused by the acidic treatment, indicating that the LbL coatings underwent phase separation and reorganisation in this situation^49^,^50^. The star-PDMAEMA extended its arms at low pH conditions, allowing more free space in the LbL coatings for the outward diffusion of IND. It is noted that the pH-sensitive morphology transitions of Coating-P/S were quite different from those of Coating-AP/S. Coating-P/S was damaged to some extent at pH 6.0, and thus we could see some LbL fragments that have aggregated into clusters on the surface. These phenomena became more obvious after five cycles of pH-challenge between pH 6.0 and pH 7.4. Coating-P/S was severely destroyed during this process. The irregular granules on its surface could be attributed to the reassembly of the oppositely charged star polymers and dendrimers. On the other hand, although there were some small holes, Coating-AP/S seemed to have restored most of its original surface morphology. All of the above *in vitro* results demonstrate that the LbL coatings would have better integrity in response to changes in pH and more stable release profiles of anti-inflammatory drugs in the case where they are anchored to PLA/HA substrates by ALN-PAMAM-COOH.

**Figure 3:**
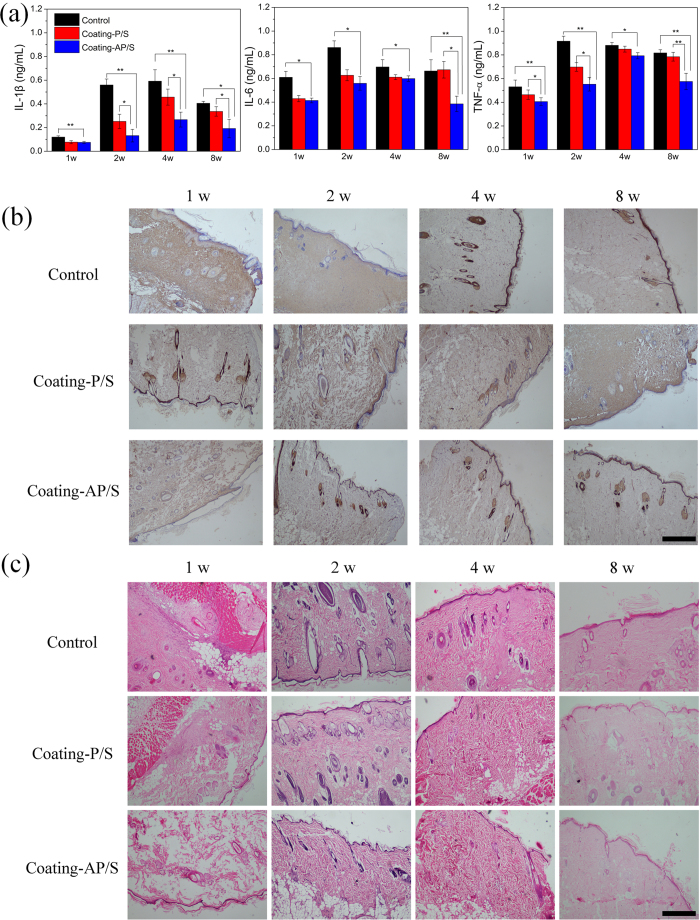
Anti-inflammatory properties of the LbL coatings after being implanted *in vivo* during 8 weeks. (**a**) Concentrations of IL-1β, IL-6, and TNF-α in Control group (naked PLA/HA), Coating-AP/S group and Coating-P/S group (n = 6, *p < 0.05, **p < 0.01). (**b**) Immunohistochemistry of TNF-α expression of surrounding tissues after the rats were sacrificed (40×, scale bar: 1 mm). (**c**) Histological analysis of surrounding tissues by H&E staining after the rats were sacrificed (40×, scale bar: 1 mm).

Materials undergo tissue response after they are implanted into living tissues. Inflammatory cytokines such as IL-1β, IL-6, and TNF-α are involved in the inflammatory process, and they can be indicators for measuring the inflammatory reaction of tissues after material implantation (Fig. 3a). Naked PLA/HA (control group) showed high levels of the three cytokines during the entire implantation period of 8 weeks. Tissues around Coating-P/S produced less cytokines than those around the control group did, with the exception of IL-6 at 8 weeks. The result suggests that IND-loaded Coating-P/S can decrease inflammation at first, but does not function well with increasing time, which is consistent with its weak stability shown in the *in vitro* experiments. Interestingly, Coating-AP/S performed well with reduced inflammation as it exhibited the least amount of inflammatory cytokines during the entire implantation period as compared with the two other groups. The statistical analysis among the different groups (the quantity of each inflammatory cytokine at each time point for analysing one factor of sample type), using one-way ANOVA, are labelled in Fig. 3a. Meanwhile, the results of two-way ANOVA show that both factors, i.e., the sample type and time point, had a statistically significant influence on the concentration of inflammatory cytokine (*p < 0.05). To visualise the concentration of inflammatory cytokines in peripheral tissues, immunohistochemical images of TNF-α expression were acquired (Fig. 3b). The brown colour indicates the amount and distribution of the expressed TNF-α. Since a lighter brown colour suggests the presence of less TNF-α, it proves that Coating-AP/S (the lightest one) provoked the least TNF-α expression during the entire 8 weeks.

The hematoxylin-eosin (H&E) staining of surrounding tissues (Fig. 3c) also shows that Coating-AP/S exhibited the least inflammatory reaction, in terms of the number of macrophages (almost none) and lymphocytes, during the entire 8 weeks. Identified by Masson’s trichrome stain, the thicknesses of the fibrous capsule were also quantified (Supplementary Table S1). As can be seen, Coating-AP/S shows the lowest thickness values among the three groups at all time points. These results collectively demonstrate that Coating-AP/S with ALN-PAMAM-COOH as the substrate-anchored layer can effectively decrease local inflammation caused by the degradation of the implanted materials. Since IND is a well-known anti-inflammatory drug, and neither star polymers nor dendrimers in the LbL coating has anti-inflammatory properties, we can infer that the reductions in inflammatory cytokine production in Coating-P/S and Coating-AP/S were indeed due to the controlled release of IND.

In the immunostained and histological images, we used the tissues surrounding the implant but did not intentionally identify the interface because we wanted to characterise the tissue response in a larger scope (especially considering that inflammatory cells can infiltrate the tissues and release inflammatory cytokines). To clearly indicate the implant-tissue interface and the presence of macrophages, we labelled them in one image as an example (Supplementary Figure S10).

Additionally, we wanted to determine whether the pH responsiveness of the coatings plays a decisive role in their *in vivo* performance. Following our previous work, by hydrolysing the star-PDMAEMA, we obtained linear PDMAEMA, which is the arm of the star polymer and does not have obvious pH responsiveness^38^. Then, we constructed a similar IND-loaded LbL coating in the form of {ALN-PAMAM-COOH/linear-PDMAEMA + (IND-loaded PAMAM-COOH/linear-PDMAEMA)_6_} (denoted as Coating-AP/L) for *in vivo* experiments. Although Coating-AP/L showed reduced inflammation (Supplementary Figure S11) compared with the control group (Fig. 3), which may have been due to the continuous leakage of IND from the coating after swelling *in vivo*, its anti-inflammatory performance was worse than that of Coating-AP/S (Fig. 3). Thus, we can say that the pH sensitivity of the star polymer-based coating is crucial for this system.

**Figure 4:**
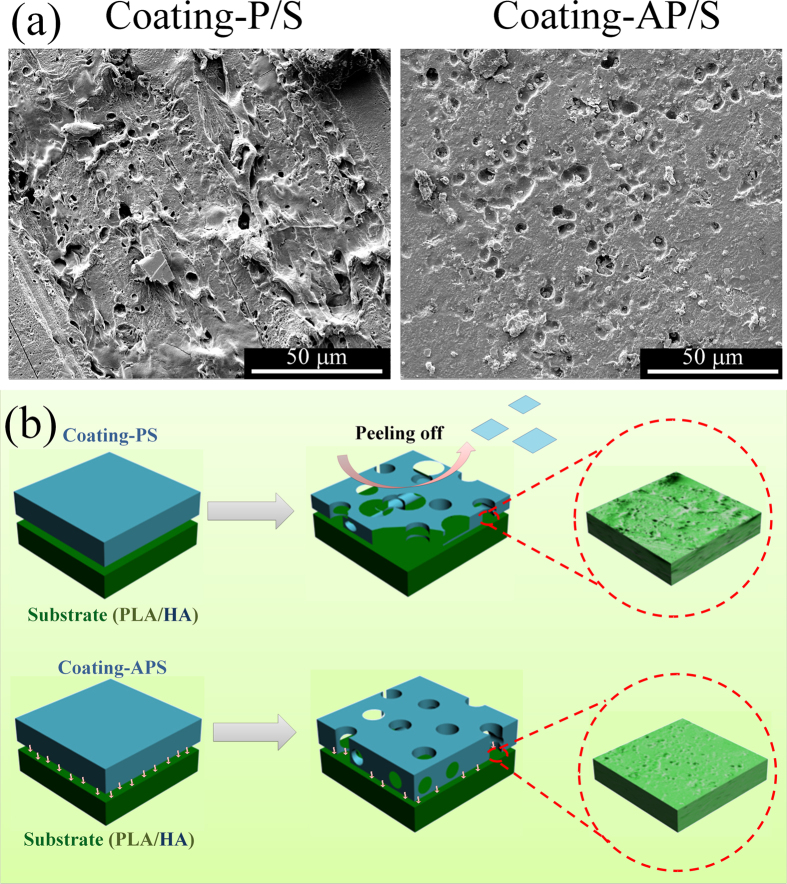
(**a**) Comparison of surface morphologies of PLA/HA substrates coated with Coating-P/S and Coanting-AP/S after being implanted for 8 weeks. (**b**) Schematic illustration of the different degradation behaviours of PLA/HA substrates coated with Coating-P/S and Coating-AP/S.

To further explore the substrate-anchored and degradation-sensitive anti-inflammatory mechanism of the LbL coatings, the surface morphologies of the PLA/HA substrates were observed by SEM after being implanted *in vivo* for different periods (Supplementary Figure S12). The naked PLA/HA and Coating-P/S both showed accelerated degradation with time going by, resulting in very rough surfaces. Coating-P/S initially exhibited a relatively flat PLA/HA surface, which gradually became rough and porous, possible due to the fast peeling off of the coating and significant substrate degradation with time. The collapse of the LbL coating and the degradation of PLA together created the coarse substrate surface. Then, the exposed surface of the PLA/HA substrate would accelerate the degradation rate and induce more inflammatory reaction. This phenomenon can explain the higher level of IL-6 at 8 weeks in Coating-P/S than that in the control group (Fig. 3a). On the contrary, with the help of ALN-PAMAM-COOH, Coating-AP/S could strongly adsorb onto the PLA/HA surface and stably respond to pH changes in the microenvironment, releasing IND to suppress local inflammation. As compared with Coating-P/S, Coating-AP/S modulated the degradation of the PLA/HA substrate in a more homogeneous manner (Fig. 4). It adsorbed tightly onto the substrate surface through a number of ALN anchors, and thus the degradation of PLA did not destroy the whole interface at the same time. The controlled homogeneous degradation is ideal for biodegradable implant materials to maintain stable functions before being completely degraded^51^,^52^.

## Conclusions

In summary, we have proposed the concept of substrate-anchored and degradation-sensitive anti-inflammatory LbL coatings for implant materials. We demonstrated that the LbL coating composed of pH-sensitive star-PDMAEMA and IND-loaded dendrimers could be immobilised onto substrate surfaces through a layer of HA-anchored ALN-PAMAM-COOH. Thus, it could effectively suppress local inflammation via controlled IND release during the degradation of the substrate for at least 8 weeks *in vivo*. Moreover, the substrate-anchored coatings modulated the degradation of the substrate in a more homogeneous manner. The novel and facile strategy therefore is opening a promising door towards the development of self-anti-inflammatory biodegradable implant materials.

## Methods

### Materials

Poly(lactic acid) (PLA, MW 106000 g/mol) under the trade name of 4032D comprising around 2% D-LA was purchased from NatureWorks (USA). Hydroxyapatite (HA, medical grade, spherical powder, 10 μm in diameter) was purchased from the National Engineering Research Center for Biomaterials, Sichuan University. PAMAM-COOH (G3.5, MW 5560 g/mol), ALN-PAMAM-COOH (G3.5, 1.6 ALN on each PAMAM-COOH, MW 5930 g/mol), fluorescein isothiocyanate (FITC)-labelled ALN-PAMAM-COOH, 21-arm star-PDMAEMA (MW 149500 g/mol) and linear PDMAEMA were prepared according to our previous reports^38^,^42^. Indomethacin (IND) was purchased from Sigma-Aldrich. All other reagents and solvents were purchased from Tianjin Bodi Chemical Holding Company and were of analytical grade. Quartz crystal microbalance (QCM) electrodes (titanium/gold crystals, resonant frequency = 5 MHz) were bought from Stanford Research System Corporation (USA). A home-built QCM, constructed using a Model QCM 100 analogue controller (Stanford Research System Corp., USA) and an Agilent 53131A universal counter, was employed for the microgravimetric experiments. Ultrapure water produced from a Millipore system with a resistivity of 18.2 MΩ.cm was used throughout the study.

### Preparation of PLA/HA substrate

PLA/HA substrates with different HA contents (0%, 5%, 10%, and 20%) were prepared. PLA was completely dissolved in dichloromethane and stirred for 48 h at room temperature to make a 10% (w/v) solution. Different amounts of HA powder were added to the solution with mechanical stirring for 24 h. After homogenisation, the mixture was incubated in an oven at 60 °C to obtain dried porous PLA/HA composite materials. These irregular materials were cut into small pieces and then thermo-compressed into round plates in a mould under conditions of 200 °C and 10 MPa for 3 min (by PS40E5ASE, Japan). The diameter and thickness of the round PLA/HA plates were 1 cm and 1 mm, respectively.

### Preparation and characterisation of IND-loaded PAMAM-COOH

An excess of IND (10 mg) was added into screw-capped vials containing 5 mL of aqueous PAMAM-COOH solution (pH 7.0) of various concentrations. This IND suspension was sonicated for 5 min and then agitated at 37 °C on an orbital shaker at 300 rpm for 48 h, after which the suspension was centrifuged at 8000 rpm for 10 min since the IND-dendrimer complex was water-soluble while IND was not. The supernatants were then filtered through a membrane filter (0.45 μm) and lyophilised to remove the water. The amount of IND loaded was measured by the decrease in IND. The structures of PAMAM-COOH, IND, and IND-loaded PAMAM-COOH were analysed by ^1^H NMR and Fourier transform infrared spectroscopy (FTIR). ^1^H NMR spectra were recorded on a Varian Unity Inova-400 spectrometer operating at 400 MHz. FTIR spectra were acquired with KBr tablets from 32 scans at 2 cm^−1^ resolution using a Nicolet 6700 FTIR spectrometer. The samples were also analysed by differential scanning calorimetry (DSC) to confirm the formation of complexation via a TA Q20 instrument. Samples were dried under vacuum at 60 °C for 24 h before analysis. Around 4 mg of each powder was filled into an aluminium pan, which was then heated at a scanning rate of 10 °C/min from 50 to 250 °C.

### Adsorption of ALN-PAMAM-COOH on the surface of PLA/HA substrate

An aqueous solution of ALN-PAMAM-COOH (1 mg mL^−1^, 200 μL) was dropped onto the surfaces of the PLA/HA substrates with different HA contents, separately. The substrates were immersed in 5 mL of deionised water for 1 h and then taken out and rinsed three times with deionised water (3 mL). The water used for substrate immersion and rinsing was collected and measured by UV to quantify the amount of ALN-PAMAM-COOH. The wavelength for UV detection was 282 nm, and the result represented the amount of ALN-PAMAM-COOH remaining in water. The amount of ALN-PAMAM-COOH adsorbed was calculated by the decrease in ALN-PAMAM-COOH in the solution. The adsorption density of ALN-PAMAM-COOH on the substrate was calculated by the amount of ALN-PAMAM-COOH adsorbed divided by the surface area of the substrate (μg mm^−2^). All experiments were run in triplicate. The FITC-labelled ALN-PAMAM-COOH solution (1 mg mL^−1^, 200 μL) was pipetted onto the surface of the PLA/HA substrates with different HA contents, separately. Deionised water (3 mL) was used to wash the sample surface three times. Thereafter, the samples were dried and observed using a fluorescence microscope (Olympus IX71) at the same magnification (40×).

### Preparation and characterisation of LbL coatings

LbL coatings were fabricated on the PLA/HA substrates with 10% HA. Aqueous solutions of ALN-PAMAM-COOH (1 mg mL^−1^, pH 6), star-PDMAEMA (1 mg mL^−1^, pH 6), PAMAM-COOH (1 mg mL^−1^, pH 6), and IND-loaded PAMAM-COOH (1 mg mL^−1^, pH 6) were prepared and then filtered through 0.45 μm Nylon Cameo membrane filters prior to use. Two groups of multilayer coatings were fabricated in the form of {PAMAM-COOH/star-PDMAEMA + (IND-loaded PAMAM-COOH/star-PDMAEMA)_6_} and {ALN-PAMAM-COOH/star-PDMAEMA + (IND-loaded PAMAM-COOH/star-PDMAEMA)_6_}, which were denoted as Coating-P/S and Coating-AP/S, respectively. The first layers of Coating-P/S and Coating-AP/S were created with 200 μL of PAMAM-COOH or ALN-PAMAM-COOH solution, respectively. The dendrimer solution was initially dropped and extended onto clean substrates for 10 min and rinsed with 1 mL of water, followed by drying with a stream of nitrogen. Then, 200 μL of the star-PDMAEMA solution was dropped and extended onto the substrates for 10 min, followed by the same rinsing and drying steps as described above. As for the following 6 bilayers, both substrates were treated alternately with 200 μL of IND-loaded PAMAM-COOH solution and 200 μL of star-PDMAEMA solution with the same process as that used for the first bilayer. UV/vis spectra were obtained with a Mapada UV-1800PC spectrophotometer. The absorbance of PAMAM-COOH at 282 nm was recorded sequentially after each adsorption step. The clean quartz substrate was initially immersed into the star-PDMAEMA (1 mg mL^−1^, pH 6) solution for 10 min and rinsed by three consecutive dips in water for 1 min during each dip. The substrate was then dried in nitrogen before performing data collection. Next, the substrate was immersed into the PAMAM-COOH (1 mg mL^−1^, pH 6) solution for 10 min, followed by the same rinsing and drying steps as described before. An LbL coating with 6 layers was obtained by alternating sequential adsorption in star-PDMAEMA and PAMAM-COOH solutions. The self-assembly process of the LbL multilayer coatings was also monitored by fabricating them on the electrode of a quartz crystal microbalance (QCM) with a frequency counter. Multilayer films were manufactured by dropping and extending 200 μL of each solution step by step onto a certain area of the QCM electrode.

### IND release *in vitro*

The *in vitro* release profile of IND from the LbL coatings was evaluated in PBS solution at 37 °C. The PLA/HA substrates with LbL coatings were immersed in 25 mL of 0.01 M PBS (pH 7.4 or 6.0). PBS (2.5 mL) was withdrawn for UV detection at different time intervals, and another 2.5 mL of fresh PBS was fed back into the original solution. All experiments were run in triplicate. The pH-sensitive release profiles of IND from the LbL coatings in response to different pH conditions were also obtained. The PLA/HA substrates with LbL coatings were immersed in PBS solution against stepwise pH changes between 6.0 and 7.4, which alternated hourly over a 10-h period. The amount of IND released during each hour was measured using 2.5 mL PBS taken from the immersion solution. All experiments were run in triplicate. The surface morphology of the LbL coatings was studied by scanning electronic microscopy (SEM) using a Hitachi S-450 (10 kV, Japan). The PLA/HA substrates with Coating-A/S and Coating-AP/S were fabricated following the previous procedure. The original coating, the coatings after incubation in PBS at pH 7.4 for 1 h, the coatings after incubation in PBS at pH 6.0 for 1 h and the coatings after 5 cycles of pH challenge between pH 6.0 and 7.4, were all observed by SEM.

### *In vivo* experiment

The *in vivo* anti-inflammatory test was carried out with male Sprague-Dawley (SD) rats. The SD rats (8 weeks old, 210–240 g) were taken care of in accordance with international standards on animal welfare, and were approved by the Animal Research Committee of Sichuan University. Each animal was anesthetised using 2.5% sodium pentobarbital throughout the implantation surgery. The control group was implanted with PLA/HA without coating. The treated groups were subcutaneously implanted with PLA/HA substrates coated with IND-loaded LbL films in the forms of Coating-P/S and Coating-AP/S, separately. The experimental period lasted for 8 weeks. Rats were sacrificed after 1, 2, 4, and 8 weeks. The implants were carefully removed and rinsed with water. There were 6 parallel samples for each group at each time point. The levels of inflammatory cytokines, i.e., IL-1β, IL-6, and TNF-α, were determined using Rat ELISA kits for interleukin-1β, interleukin-6, and tumour necrosis factor-α, respectively (R&D, USA). For each sample, 1 g of subcutaneous tissues adjacent to the LbL coatings was cut into fragments, suspended in normal saline (1 mL), and homogenised with a homogeniser. The homogenised solution was taken out and centrifuged (5000 rpm) for 20 min. The supernatant samples were frozen at −80 °C until analysis. The samples were thawed and processed to evaluate the concentrations of IL-1β, IL-6, and TNF-α with protocols suggested by the manufacturer using a micro plate reader (Spectra Plus, Tecan, Zurich, Switzerland). The results were statistically analysed by both one-way ANOVA (the quantity of each inflammatory cytokine at each time for analysing one factor of sample type) and two-way ANOVA (for analysing two factors, namely the sample type and the time), and a p-value of < 0.05 was considered to be statistically significant. The expression of TNF-α in the tissue was also detected by immunohistochemical StreptAvidin-Biotin Complex (SABC) staining. Moreover, the subcutaneous tissues surrounding the films were stained by hematoxylin-eosin (H&E). All images were taken using an optical microscope equipped with a colour camera at 40× magnification. Tissue samples were also subjected to Masson’s trichrome staining to identify fibrosis and to characterise capsule thickness (6 random sites for each sample). During the 8 weeks of implantation, the PLA/HA tablets with or without LbL coatings were taken out and rinsed with water immediately. The surface morphology of the LbL films was observed using SEM. Coating-AP/L constructed from linear PDMAEMA instead of star polymers was also studied for comparison.

## Additional Information

**How to cite this article**: Wu, D. *et al.* Substrate-anchored and degradation-sensitive anti-inflammatory coatings for implant materials. *Sci. Rep.* **5**, 11105; doi: 10.1038/srep11105 (2015).

## Supplementary Material

Supplementary Information

## Figures and Tables

**Figure 1:**
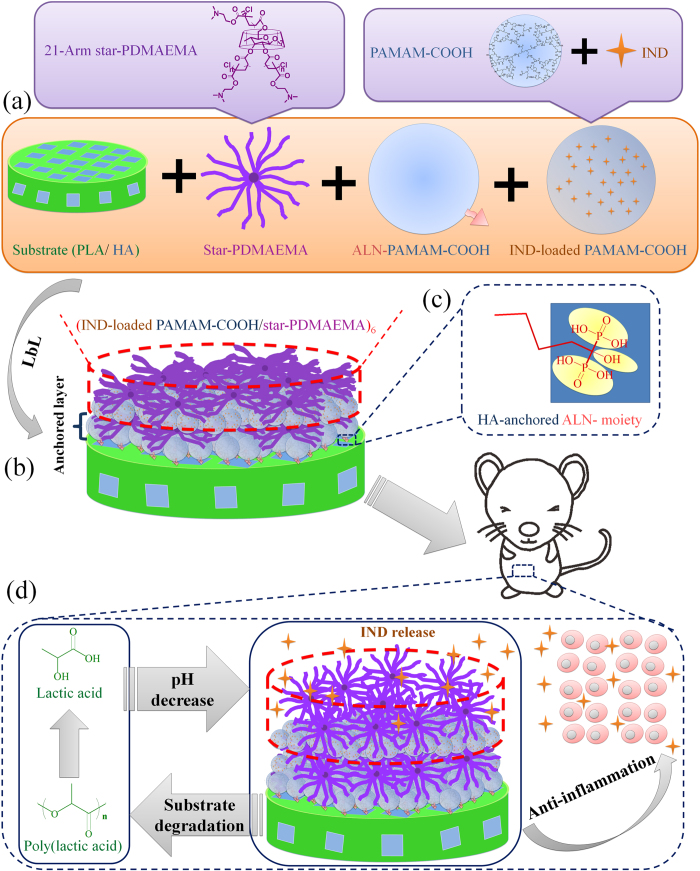
Schematic illustration of the substrate-anchored and degradation-sensitive anti-inflammatory coating for implant materials. (**a**) Biodegradable substrate of implant material (PLA/HA) and building blocks of the LbL coating (star-PDMAEMA, ALN-PAMAM-COOH and IND-loaded PAMAM-COOH); (**b**) Coating-AP/S in the form of {ALN-PAMAM-COOH/star-PDMAEMA+(IND-loaded PAMAM-COOH/star-PDMAEMA)_6_}; (**c**) Substrate-anchored property provided by the ALN moiety; (**d**) Degradation-sensitive anti-inflammation: PLA degrades into acidic products and causes pH decrease, which will induce star-PDMAEMA to be fully stretched and rearrange the LbL coating to accelerate the release of anti-inflammatory drug (IND).

## References

[b1] Anderson J. M. Biological responses to materials. Ann. Rev. Mater. Res. 31, 81–110 (2001).

[b2] Benkirane-Jessel N. *et al.* Control of monocyte morphology on and response to model surfaces for implants equipped with anti-inflammatory agents. Adv. Mater. 16, 1507–1511 (2004).

[b3] He W., McConnell G. C., Schneider T. M. & Bellamkonda R. V. A novel anti-inflammatory surface for neural electrodes. Adv. Mater. 19, 3529–3533 (2007).

[b4] Cobelli N., Scharf B., Crisi G. M., Hardin J. & Santambrogio L. Mediators of the inflammatory response to joint replacement devices. Nature Rev. Rheumatol. 7, 600–608 (2011). 21894210 10.1038/nrrheum.2011.128

[b5] Franz S., Rammelt S., Scharnweber D. & Simon J. C. Immune responses to implants - a review of the implications for the design of immunomodulatory biomaterials. Biomaterials 32, 6692–6709 (2011). 21715002 10.1016/j.biomaterials.2011.05.078

[b6] Velard F., Braux J., Amedee J. & Laquerriere P. Inflammatory cell response to calcium phosphate biomaterial particles: an overview. Acta Biomater. 9, 4956–4963 (2013). 23036944 10.1016/j.actbio.2012.09.035

[b7] Zhang H. N. *et al.* Tissue-compliant neural implants from microfabricated carbon nanotube multilayer composite. Acs Nano 7, 7619–7629 (2013). 23930825 10.1021/nn402074y

[b8] Hench L. L. & Polak J. M. Third-generation biomedical materials. Science 295, 1014–1017 (2002). 11834817 10.1126/science.1067404

[b9] Athanasiou K. A., Niederauer G. G. & Agrawal C. M. Sterilization, toxicity, biocompatibility and clinical applications of polylactic acid polyglycolic acid copolymers. Biomaterials 17, 93–102 (1996). 8624401 10.1016/0142-9612(96)85754-1

[b10] Bostman O. & Pihlajamaki H. Clinical biocompatibility of biodegradable orthopaedic implants for internal fixation: a review. Biomaterials 21, 2615–2621 (2000). 11071611 10.1016/s0142-9612(00)00129-0

[b11] Ruiz-Hitzky E., Darder M., Aranda P. & Ariga K. Advances in biomimetic and nanostructured biohybrid materials. Adv. Mater. 22, 323–336 (2010). 20217713 10.1002/adma.200901134

[b12] Curtin C. M. *et al.* Innovative collagen nano-hydroxyapatite scaffolds offer a highly efficient non-viral gene delivery platform for stem cell-mediated bone formation. Adv. Mater. 24, 749–754 (2012). 22213347 10.1002/adma.201103828

[b13] Shah N. J., Hong J., Hyder M. N. & Hammond P. T. Osteophilic multilayer coatings for accelerated bone tissue growth. Adv. Mater. 24, 1445–1450 (2012). 22311551 10.1002/adma.201104475 PMC3870474

[b14] Li C. *et al.* Amyloid-hydroxyapatite bone biomimetic composites. Adv. Mater. 26, 3207–3212 (2014). 24634054 10.1002/adma.201306198

[b15] Olszta M. J. *et al.* Bone structure and formation: a new perspective. Mater. Sci. Eng. R 58, 77–116 (2007).

[b16] Rizzi S. C. *et al.* Biodegradable polymer/hydroxyapatite composites: surface analysis and initial attachment of human osteoblasts. J. Biomed. Mater. Res. 55, 475–486 (2001). 11288075 10.1002/1097-4636(20010615)55:4<475::aid-jbm1039>3.0.co;2-q

[b17] Hu Y. Y., Zhang C., Zhang S. M., Xiong Z. & Xu J. Q. Development of a porous poly(L-lactic acid)/hydroxyapatite/collagen scaffold as a BMP delivery system and its use in healing canine segmental bone defect. J. Biomed .Mater. Res. A 67A, 591–598 (2003). 10.1002/jbm.a.10070 14566802

[b18] Liao S. S., Cui F. Z., Zhang W. & Feng Q. L. Hierarchically biomimetic bone scaffold materials: nano-HA/collagen/PLA composite. J. Biomed. Mater. Res. B 69, 158–165 (2004). 10.1002/jbm.b.20035 15116405

[b19] Kothapalli C. R., Shaw M. T. & Wei M. Biodegradable HA-PLA 3-D porous scaffolds: effect of nano-sized filler content on scaffold properties. Acta Biomater. 1, 653–662 (2005). 16701846 10.1016/j.actbio.2005.06.005

[b20] Kaito T. *et al.* Potentiation of the activity of bone morphogenetic protein-2 in bone regeneration by a PLA-PEG/hydroxyapatite composite. Biomaterials 26, 73–79 (2005). 15193882 10.1016/j.biomaterials.2004.02.010

[b21] Shen L., Yang H., Ying J., Qiao F. & Peng M. Preparation and mechanical properties of carbon fiber reinforced hydroxyapatite/polylactide biocomposites. J. Mater. Sci. Mater. M 20, 2259–2265 (2009). 19488680 10.1007/s10856-009-3785-2

[b22] Kutikov A. B. & Song J. An amphiphilic degradable polymer/hydroxyapatite composite with enhanced handling characteristics promotes osteogenic gene expression in bone marrow stromal cells. Acta Biomater. 9, 8354–8364 (2013). 23791675 10.1016/j.actbio.2013.06.013 PMC3745304

[b23] Ding C. *et al.* Regeneration of a goat femoral head using a tissue-specific, biphasic scaffold fabricated with CAD/CAM technology. Biomaterials 34, 6706–6716 (2013). 23773816 10.1016/j.biomaterials.2013.05.038

[b24] Matsusaki M. & Akashi M. Novel functional biodegradable polymer IV: pH-Sensitive controlled release of fibroblast growth factor-2 from a poly(gamma-glutamic acid)-sulfonate matrix for tissue engineering. Biomacromolecules 6, 3351–3356 (2005). 16283765 10.1021/bm050369m

[b25] Ganta S., Devalapally H., Shahiwala A. & Amiji M. A review of stimuli-responsive nanocarriers for drug and gene delivery. J. Control. Release 126, 187–204 (2008). 18261822 10.1016/j.jconrel.2007.12.017

[b26] Wang C., Javadi A., Ghaffari M. & Gong S. A pH-sensitive molecularly imprinted nanospheres/hydrogel composite as a coating for implantable biosensors. Biomaterials 31, 4944–4951 (2010). 20346500 10.1016/j.biomaterials.2010.02.073

[b27] Boudou T., Crouzier T., Ren K., Blin G. & Picart C. Multiple functionalities of polyelectrolyte multilayer films: new biomedical applications. Adv. Mater. 22, 441–467 (2010). 20217734 10.1002/adma.200901327

[b28] Hammond P. T. Building biomedical materials layer-by-layer. Mater. Today 15, 196–206 (2012).

[b29] DeMuth P. C. *et al.* Polymer multilayer tattooing for enhanced DNA vaccination. Nature Mater. 12, 367–376 (2013). 23353628 10.1038/nmat3550 PMC3965298

[b30] Sato K., Yoshida K., Takahashi S. & Anzai J. pH- and sugar-sensitive layer-by-layer films and microcapsules for drug delivery. Adv. Drug Deliver. Rev. 63, 809–821 (2011). 10.1016/j.addr.2011.03.015 21510988

[b31] Wang J. F. *et al.* Self-assembled multilayer films based on dendrimers with covalent interlayer linkage. Chem. Mater. 14, 2854–2858 (2002).

[b32] Khopade A. J. & Caruso F. Electrostatically assembled polyelectrolyte/dendrimer multilayer films as ultrathin nanoreservoirs. Nano Lett. 2, 415–418 (2002).

[b33] Connal L. A. *et al.* PH-responsive poly(acrylic acid) core cross-linked star polymers: Morphology transitions in solution and multilayer thin films. Macromolecules 41, 2620–2626 (2008).

[b34] Tomita S., Sato K. & Anzai J. I. Layer-by-layer assembled thin films composed of carboxyl-terminated poly(amidoamine) dendrimer as a pH-sensitive nano-device. J. Colloid Interf. Sci. 326, 35–40 (2008). 10.1016/j.jcis.2008.06.054 18649890

[b35] Kim B. S., Gao H. F., Argun A. A., Matyjaszewski K. & Hammond P. T. All-star polymer multilayers as pH-responsive nanofilms. Macromolecules 42, 368–375 (2009).

[b36] Kim Y., Kook K., Hwang S. K., Park C. & Cho J. Polymer/perovskite-type nanoparticle multilayers with multielectric properties prepared from ligand addition-induced layer-by-layer assembly. Acs Nano 8, 2419–2430 (2014). 24571293 10.1021/nn405988d

[b37] Guo Z. *et al.* Effect of molecular weight and arm number on the growth and pH-dependent morphology of star poly[2-(dimethylamino)ethyl methacrylate]/poly(styrenesulfonate) multilayer films. Macromolecules 43, 9087–9093 (2010).

[b38] Chen X., Wu W., Guo Z., Xin J. & Li J. Controlled insulin release from glucose-sensitive self-assembled multilayer films based on 21-arm star polymer. Biomaterials 32, 1759–1766 (2011). 21112085 10.1016/j.biomaterials.2010.11.002

[b39] Chen X. *et al.* The influence of arrangement sequence on the glucose-responsive controlled release profiles of insulin-incorporated LbL films. Acta Biomater. 8, 4380–4388 (2012). 22902821 10.1016/j.actbio.2012.08.014

[b40] Luo J. *et al.* Super long-term glycemic control in diabetic rats by glucose-sensitive LbL films constructed of supramolecular insulin assembly. Biomaterials 33, 8733–8742 (2012). 22954517 10.1016/j.biomaterials.2012.08.041

[b41] Wu W., Wang W. & Li J. Star polymers: Advances in biomedical applications. Prog. Polym. Sci. http://dx.doi.org/10.1016/j.progpolymsci.2015.02.002 (2015).

[b42] Wu D. *et al.* Hydroxyapatite-anchored dendrimer for in situ remineralization of human tooth enamel. Biomaterials 34, 5036–5047 (2013). 23578556 10.1016/j.biomaterials.2013.03.053

[b43] Yang X., Shang H., Ding C. & Li J. Recent developments and applications of bioinspired dendritic polymers. Polym. Chem. 6, 668–680 (2015).

[b44] Jang W. D., Kamruzzaman Selim K. M., Lee C. H. & Kang I. K. Bioinspired application of dendrimers: from bio-mimicry to biomedical applications. Prog. Polym. Sci. 34, 1–23 (2009).

[b45] Zhou Y., Huang W., Liu J., Zhu X. & Yan D. Self-assembly of hyperbranched polymers and its biomedical applications. Adv. Mater. 22, 4567–4590 (2010). 20853374 10.1002/adma.201000369

[b46] Mintzer M. A. & Grinstaff M. W. Biomedical applications of dendrimers: a tutorial. Chem. Soc. Rev. 40, 173–190 (2011). 20877875 10.1039/b901839p

[b47] Zhou Y. *et al.* Triclosan-loaded poly(amido amine) dendrimer for simultaneous treatment and remineralization of human dentine. Colloid. Surface. B. 115, 237–243 (2014). 10.1016/j.colsurfb.2013.11.045 24362062

[b48] Tokarev I. & Minko S. Multiresponsive, hierarchically structured membranes: new, challenging, biomimetic materials for biosensors, controlled release, biochemical gates, and nanoreactors. Adv. Mater. 21, 241–247 (2009).

[b49] Mendelsohn J. D. *et al.* Fabrication of microporous thin films from polyelectrolyte multilayers. Langmuir 16, 5017–5023 (2000).

[b50] Lutkenhaus J. L., McEnnis K. & Hammond P. T. Nano- and microporous layer-by-layer assemblies containing linear poly(ethylenimine) and poly(acrylic acid). Macromolecules 41, 6047–6054 (2008).

[b51] Wang Y. *et al.* In vivo degradation of three-dimensional silk fibroin scaffolds. Biomaterials 29, 3415–3428 (2008). 18502501 10.1016/j.biomaterials.2008.05.002 PMC3206261

[b52] Xi T. *et al.* In vitro and in vivo changes to PLGA/sirolimus coating on drug eluting stents. Biomaterials 31, 5151–5158 (2010). 20382420 10.1016/j.biomaterials.2010.02.003

