# Supplementary material for: Substrate-anchored and degradation-sensitive anti-inflammatory coatings for implant materials
Source: Sci Rep. 2015 Jun 16;5:11105. doi: 10.1038/srep11105 (PMC4650647; doi:10.1038/srep11105)
Supplement: Supplementary Information [file srep11105-s1.pdf]

Supplementary Information

Substrate-anchored and degradation-sensitive anti-inflammatory coatings for implant materials

Duo Wu[1,†], *Xingyu Chen*[1,†], *Tianchan Chen*[1], *Chunmei Ding*[1], *Wei Wu*[1], *and Jianshu Li*[1,2,]*

[1] College of Polymer Science and Engineering, Sichuan University, Chengdu 610065, China

[2] State Key Laboratory of Polymer Materials Engineering, Sichuan University, Chengdu 610065, China

* Corresponding author: jianshu_li@scu.edu.cn

[†] These authors contributed equally to this work.

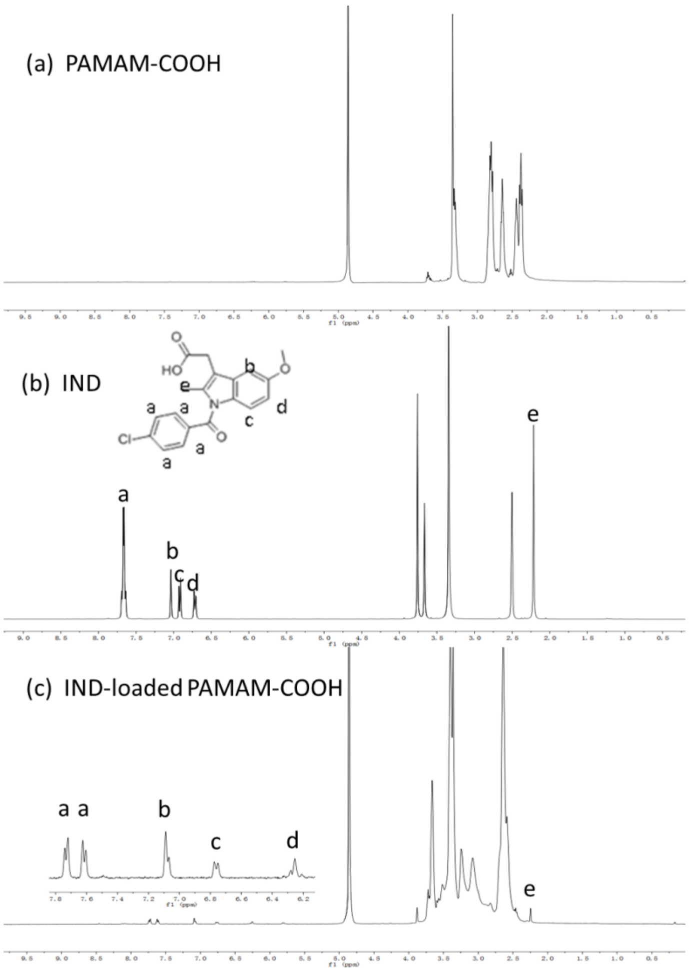

Supplementary Figure S1. ^{1}H NMR spectra of PAMAM-COOH (a), IND (b) and IND-loaded PAMAM-COOH (c). The solvents were D_2O for (a) and (c), while DMSO-d_6 for (b).

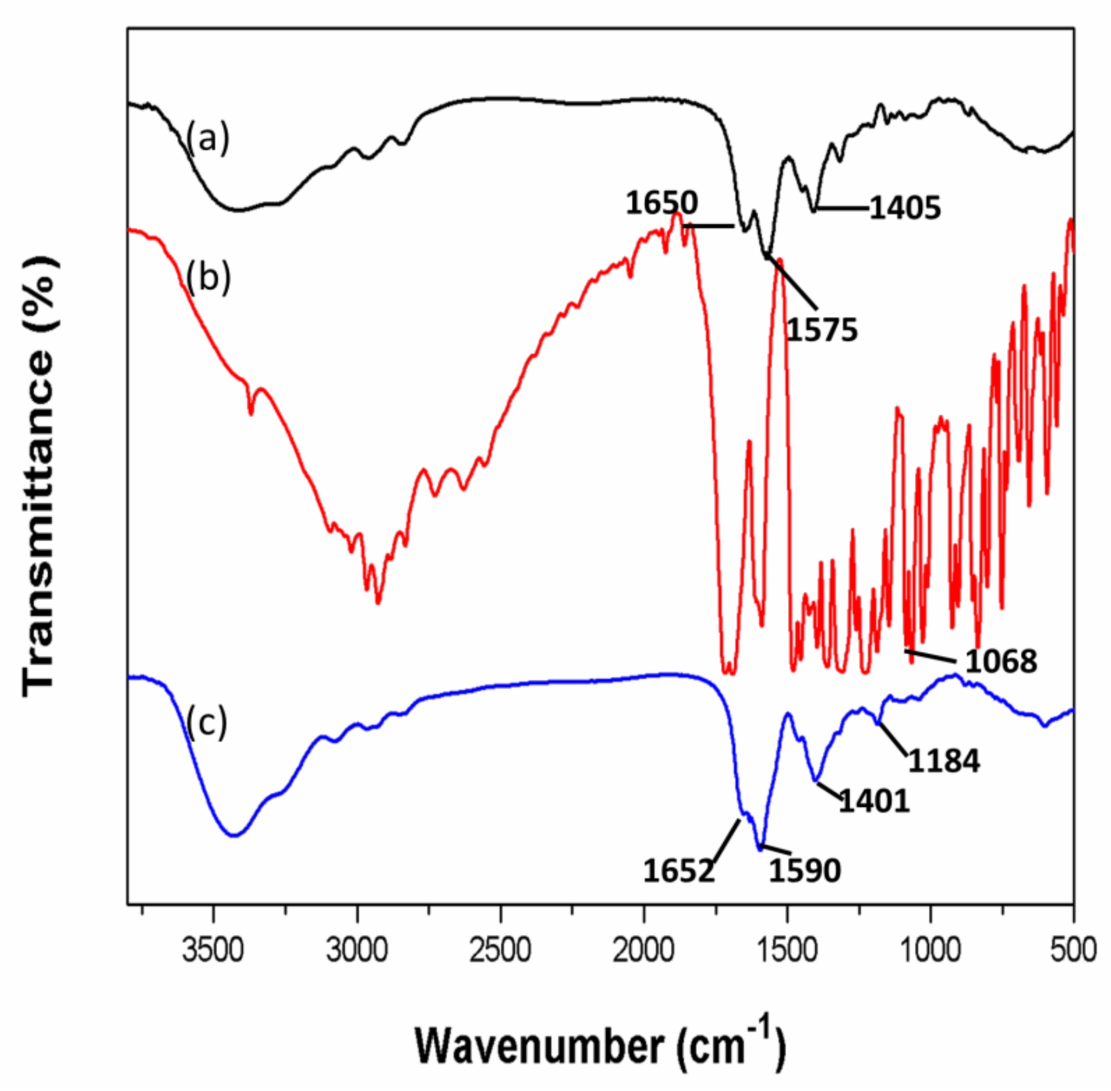

Supplementary Figure S2. FTIR spectra of PAMAM-COOH (a), IND (b), and IND-loaded PAMAM-COOH (c) recorded from KBr tablets.

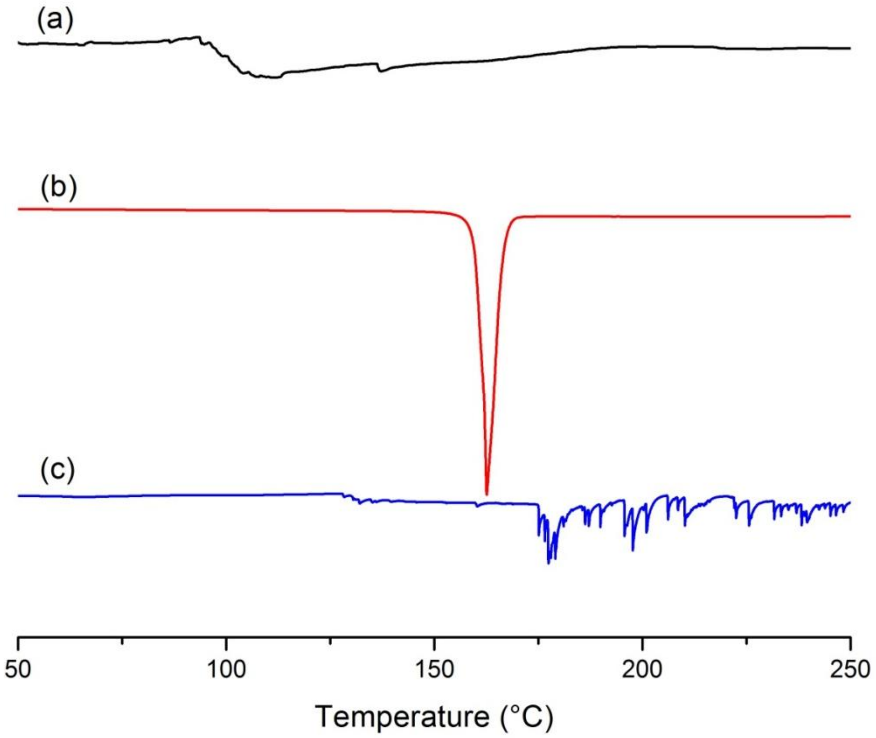

Supplementary Figure S3. DSC curves of PAMAM-COOH (a), IND (b), and IND-loaded PAMAM-COOH (c).

The endothermic melting peak of IND corresponding to Form I polymorph at around 162.0 ℃ is disappeared in the curve of IND-loaded PAMAM-COOH. It proves that the crystal structure of IND has been completely destroyed by forming complexation with PAMAM-COOH. In addition, several endothermal peaks are observed for the IND-loaded PAMAM-COOH complex, all of which are higher than 175 ℃. These peaks are not corresponding to peaks presented neither for PAMAM-COOH nor for IND alone, but should be due to the gradual decomposition of the IND-dendrimer complex.

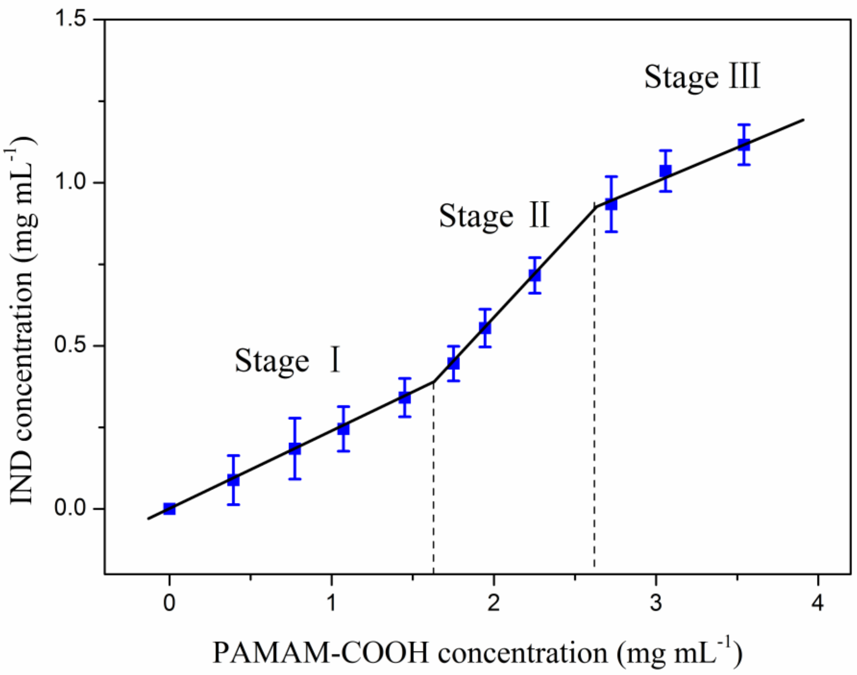

Supplementary Figure S4. Aqueous solubility of IND in the presence of PAMAM-COOH at different concentrations.

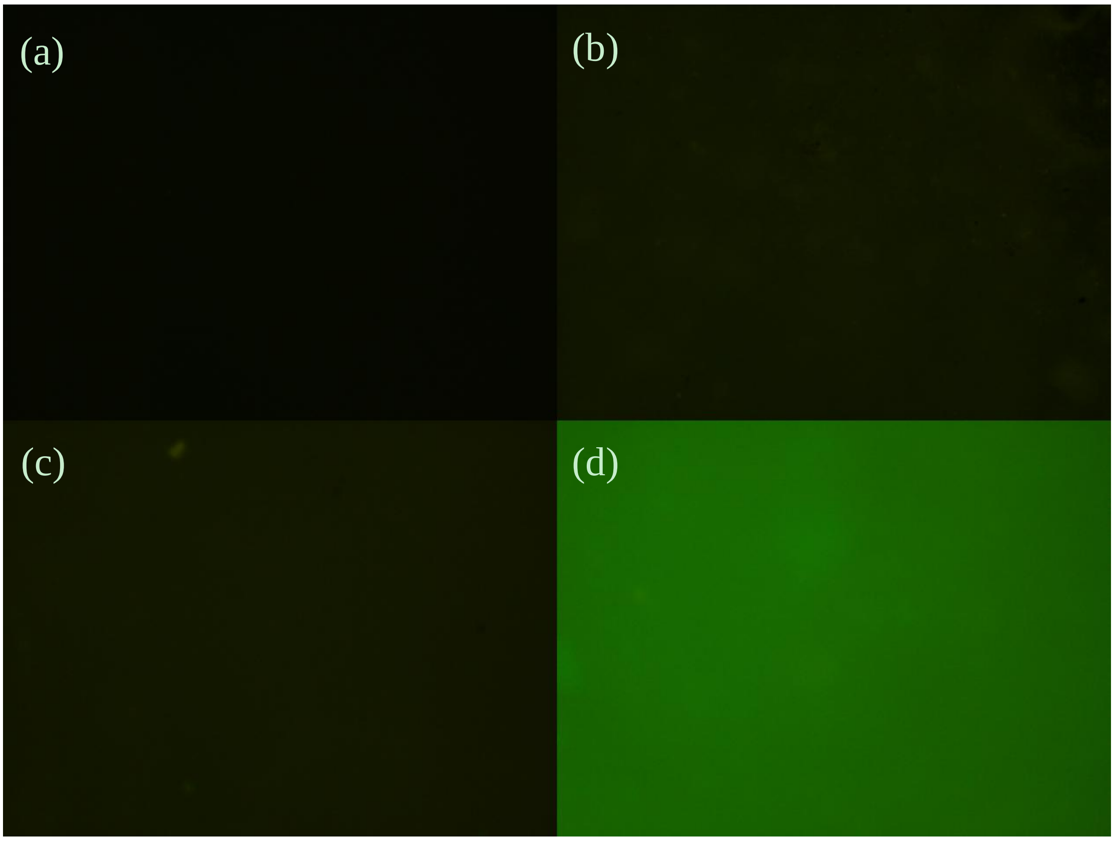

Supplementary Figure S5. Fluorescence microscope images of FITC-labeled ALN-PAMAM-COOH adsorbed onto the PLA/HA substrates with different HA contents: 0% (a), 5% (b), 10% (c), and 20% (d).

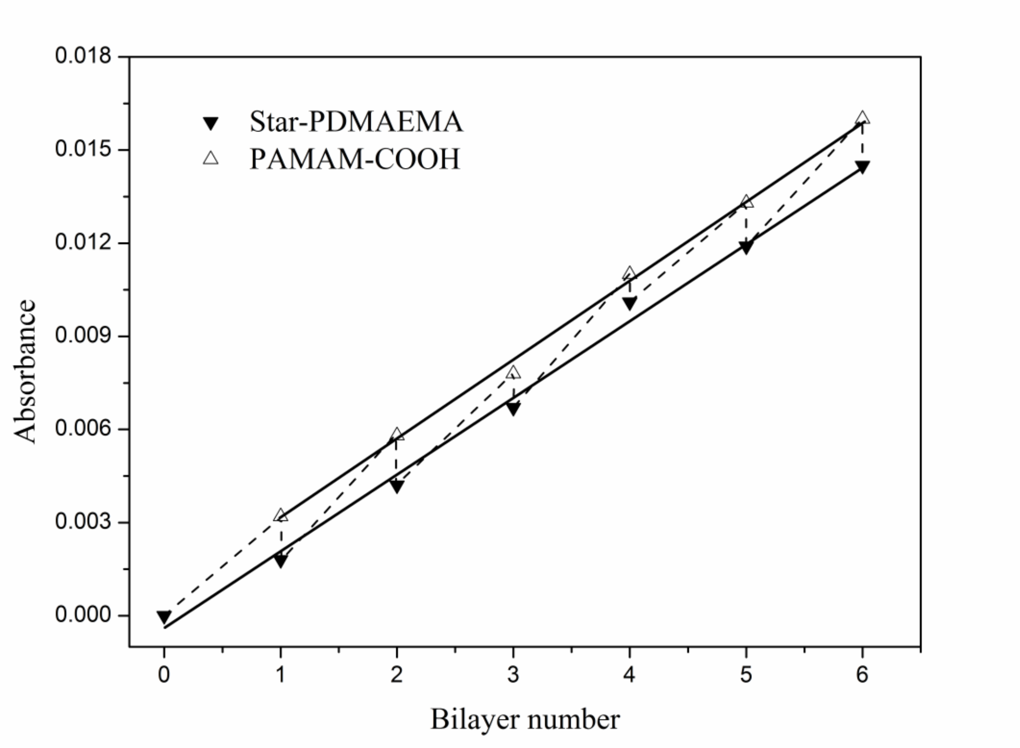

Supplementary Figure S6. Absorbance of PAMAM-COOH at 282 nm versus the number of star PDMAEMA/PAMAM-COOH bilayers. The absorbance of the first layer (star-PDMAEMA) deposited on quartz substrate corresponds to zero. The dotted line indicates the stepwise PAMAM-COOH adsorption/extraction process.

It has been reported that PDMAEMA could be well-covered on silicon wafer. Therefore, star-PDMAEMA, instead of PAMAM-COOH, were deposited on the quartz surface as the first layer in this experiment. The maximum absorption of PAMAM-COOH at 282 nm was chosen in order to avoid the interferer adsorption of star-PDMAEMA between 200 and 240 nm.

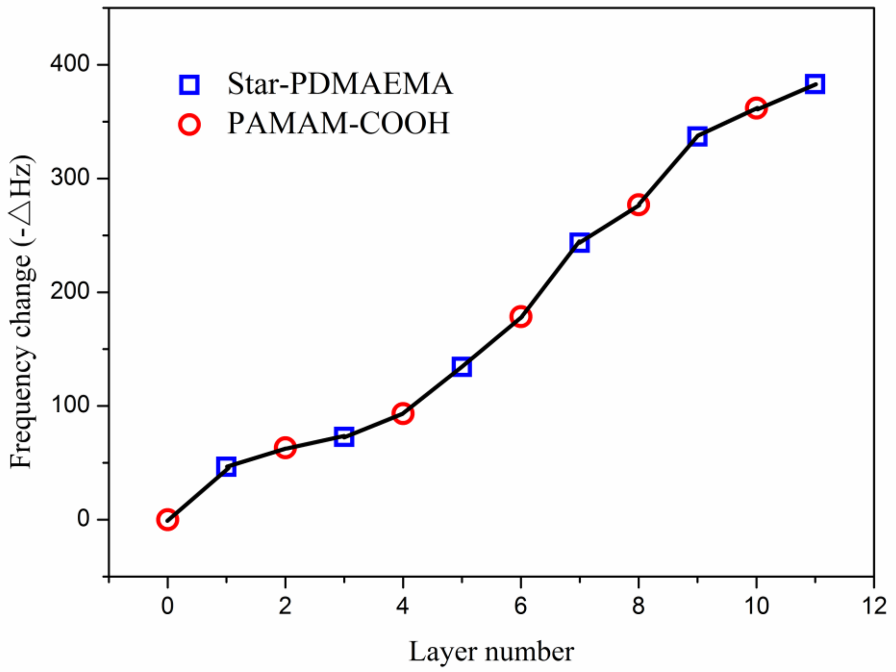

Supplementary Figure S7. QCM frequency changes as a function of layer number for star-PDMAEMA/PAMAM-COOH bilayers. Each solution was prepared as 1 mg mL^{-1} at pH 6.

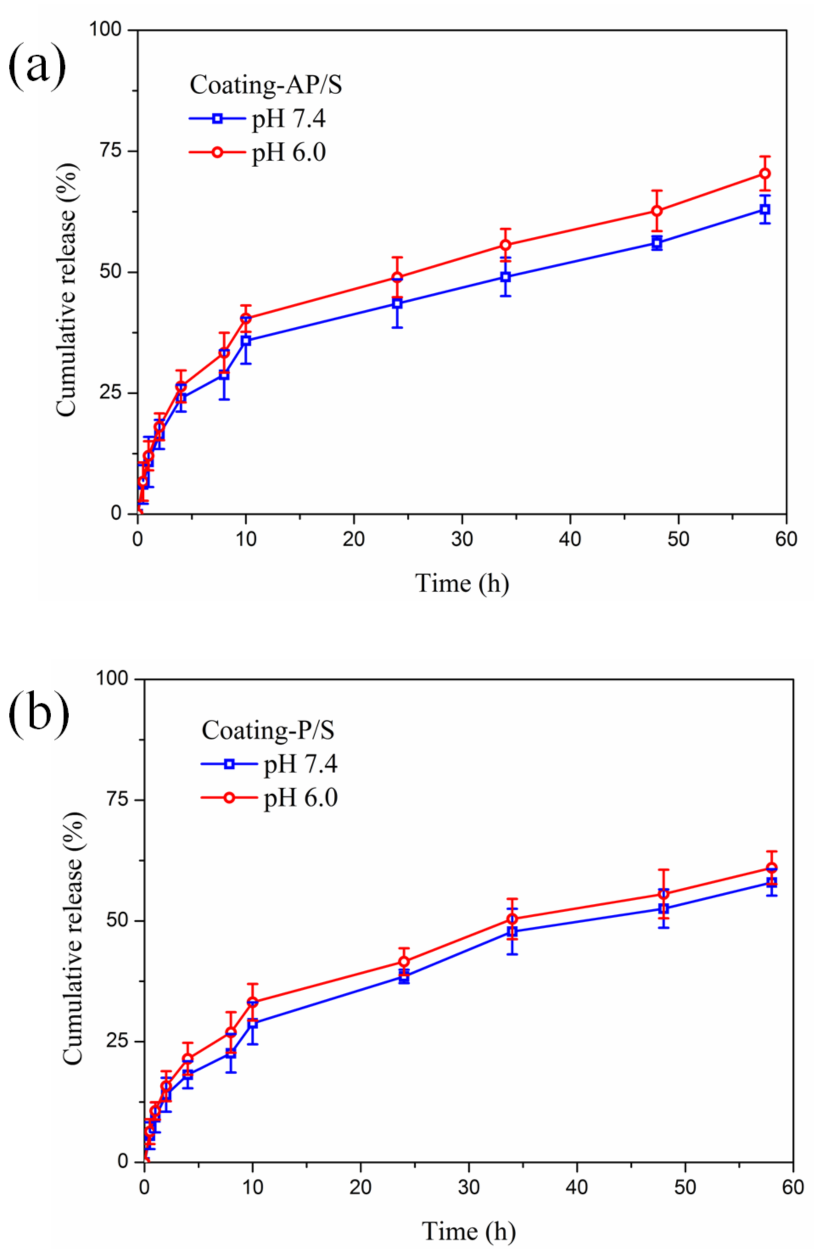

Supplementary Figure S8. Release profiles of IND from Coating-AP/S (a) and Coating-P/S (b) at pH 7.4 and 6.0 (n=3).

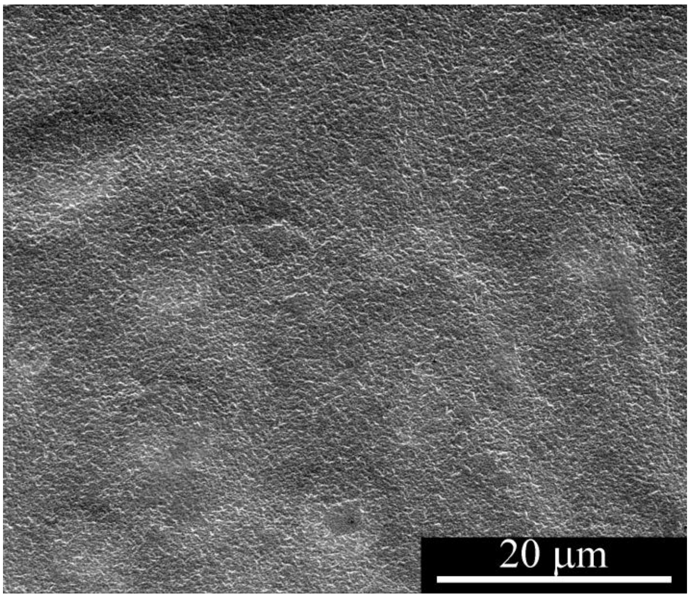

Supplementary Figure S9. SEM image of the LbL coating surface after fabrication (Coating-AP/S).

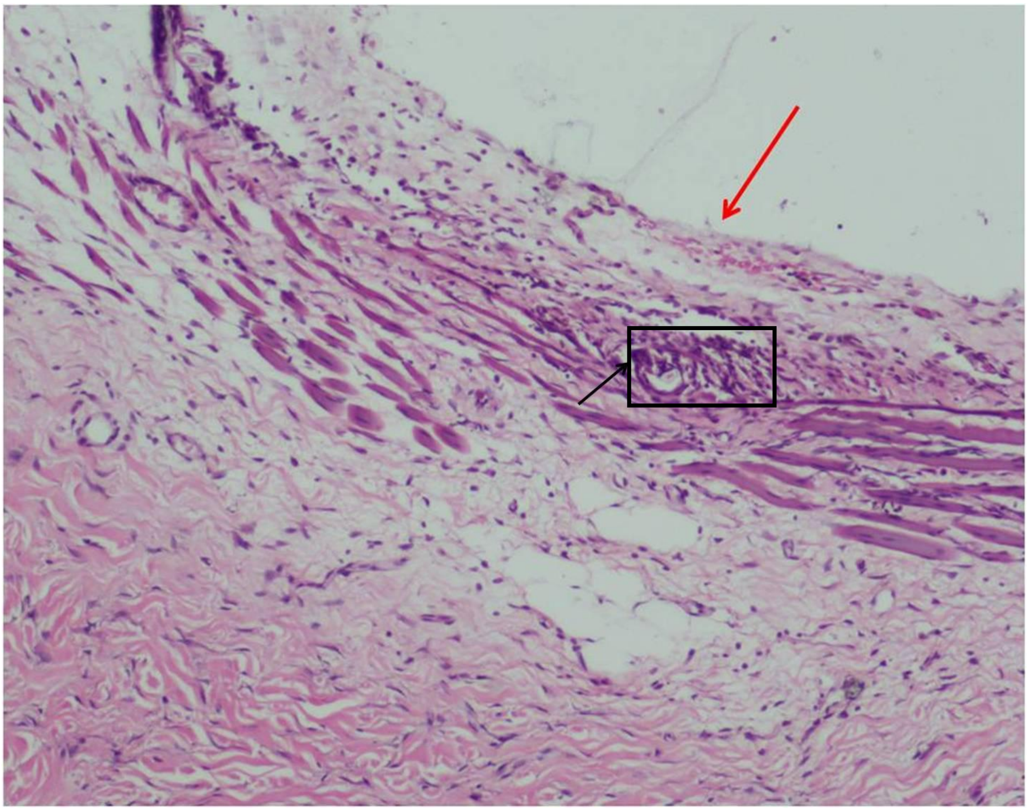

Supplementary Figure S10. Histological analysis of surrounding tissues (Control sample, 2 w) by H&E staining after the rats were sacrificed to indicate the implant-tissue interface (red-arrow) and macrophages (black-arrow) (100×).

(a)

	Concentrations of inflammatory cytokines (ng/mL)		
	1 w	2 w	4 w
IL-1β	0.092 ±0.054	0.436 ±0.075	0.512 ±0.084
IL-6	0.583 ±0.051	0.746 ±0.044	0.632 ±0.075
TNF-α	0.496 ±0.046	0.825 ±0.023	0.841 ±0.056

(b)

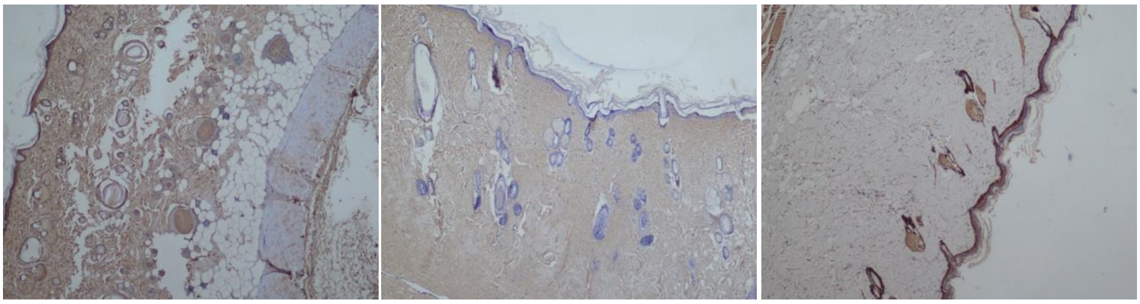

1w 2w 4w

(c)

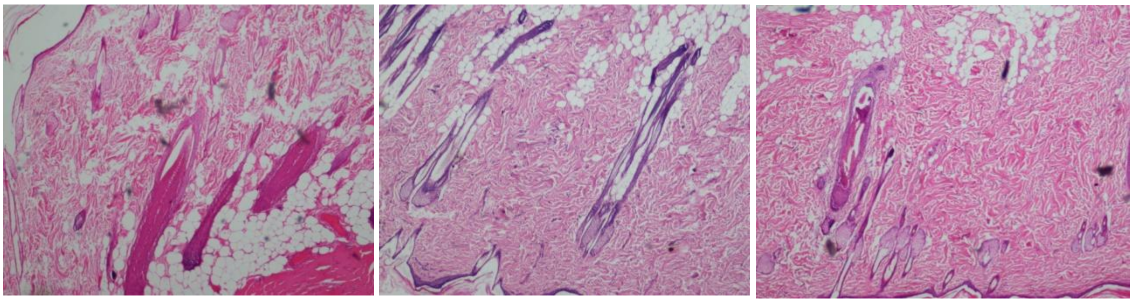

1w 2w 4w

Supplementary Figure S11. Anti-inflammatory properties of Coating-AP/L after being implanted *in vivo*. (a) Concentrations of IL-1β, IL-6, and TNF-α. (b) Immunohistochemistry of TNF-α expression of surrounding tissues after the rats were sacrificed (40×). (c) Histological analysis of surrounding tissues by H&E staining after the rats were sacrificed (40×).

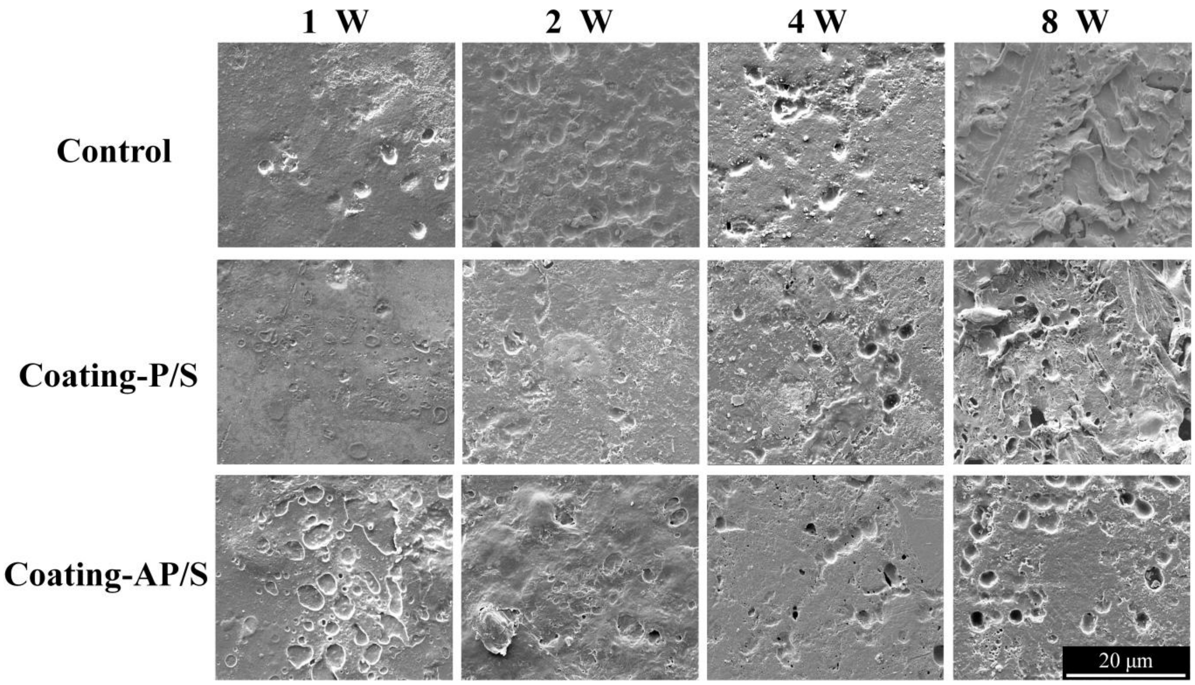

Supplementary Figure S12. SEM images of the surface morphologies of PLA/HA substrates after being implanted *in vivo*.

Supplementary Table S1. The capsule thickness of implant tablets at different time points.

Sample	Thickness of fibrous capsule around the implant tablets (μm)		
	2 w	4 w	8 w
Control	192.31 ± 7.05	213.47 ± 5.37	216.29 ± 4.58
Coating-P/S	156.22 ± 1.45	163.19 ± 8.54	164.44 ± 3.17
Coating-AP/S	107.58 ± 6.34	109.87 ± 5.22	103.34 ± 2.35
